# Cutaneous exposure to agglomerates of silica nanoparticles and allergen results in IgE-biased immune response and increased sensitivity to anaphylaxis in mice

**DOI:** 10.1186/s12989-015-0095-3

**Published:** 2015-06-26

**Authors:** Toshiro Hirai, Yasuo Yoshioka, Hideki Takahashi, Ko-ichi Ichihashi, Asako Udaka, Takahide Mori, Nobuo Nishijima, Tokuyuki Yoshida, Kazuya Nagano, Haruhiko Kamada, Shin-ichi Tsunoda, Tatsuya Takagi, Ken J. Ishii, Hiromi Nabeshi, Tomoaki Yoshikawa, Kazuma Higashisaka, Yasuo Tsutsumi

**Affiliations:** Laboratory of Toxicology and Safety Science, Graduate School of Pharmaceutical Sciences, Osaka University, 1-6 Yamadaoka, Suita, Osaka 565-0871 Japan; Vaccine Creation Project, BIKEN Innovative Vaccine Research Alliance Laboratories, Research Institute for Microbial Diseases, Osaka University, 3-1 Yamadaoka, Suita, Osaka 565-0871 Japan; BIKEN Center for Innovative Vaccine Research and Development, The Research Foundation for Microbial Diseases of Osaka University, 3-1 Yamadaoka, Suita, Osaka 565-0871 Japan; Laboratory of Innovative Antibody Engineering and Design, Center for Drug Innovation and Screening, National Institute of Biomedical Innovation, 7-6-8 Saitoasagi, Ibaraki, Osaka 567-0085 Japan; Laboratory of Biopharmaceutical Research, National Institute of Biomedical Innovation, 7-6-8 Saitoasagi, Ibaraki, Osaka 567-0085 Japan; The Center for Advanced Medical Engineering and Informatics, Osaka University, 1-6 Yamadaoka, Suita, Osaka 565-0871 Japan; Laboratory of Environmental Pharmacometrics, Graduate School of Pharmaceutical Sciences, Osaka University, 1-6 Yamadaoka, Suita, Osaka 565-0871 Japan; Genome Information Research Center, Research Institute for Microbial Diseases, Osaka University, 3-1 Yamadaoka, Suita, Osaka 565-0871 Japan; Laboratory of Adjuvant Innovation, National Institute of Biomedical Innovation, 7-6-8 Saitoasagi, Ibaraki, Osaka 567-0085 Japan; Laboratory of Vaccine Science, Immunology Frontier Research Center, World Premier International Research Center, Osaka University, 3-1 Suita, Osaka, 565-0871 Japan; Division of Foods, National Institute of Health Sciences, 1-18-1 Kamiyoga, Setagaya-ku, Tokyo, 158-8501 Japan

**Keywords:** Atopic dermatitis, Agglomerate, Aggregate, Anaphylaxis, Blocking antibody, IgE, IgG, Mite, Nanomaterials, Nanoparticles, Particulate matter

## Abstract

**Background:**

The skin is a key route of human exposure to nanomaterials, which typically occurs simultaneously with exposure to other chemical and environmental allergen. However, little is known about the hazards of nanomaterial exposure via the skin, particularly when accompanied by exposure to other substances.

**Results:**

Repeated topical treatment of both ears and the shaved upper back of NC/Nga mice, which are models for human atopic dermatitis (AD), with a mixture of mite extract and silica nanoparticles induced AD-like skin lesions. Measurements of ear thickness and histologic analyses revealed that cutaneous exposure to silica nanoparticles did not aggravate AD-like skin lesions. Instead, concurrent cutaneous exposure to mite allergens and silica nanoparticles resulted in the low-level production of allergen-specific IgGs, including both the Th2-related IgG1 and Th1-related IgG2a subtypes, with few changes in allergen-specific IgE concentrations and in Th1 and Th2 immune responses. In addition, these changes in immune responses increased the sensitivity to anaphylaxis. Low-level IgG production was induced when the mice were exposed to allergen–silica nanoparticle agglomerates but not when the mice exposed to nanoparticles applied separately from the allergen or to well-dispersed nanoparticles.

**Conclusions:**

Our data suggest that silica nanoparticles themselves do not directly affect the allergen-specific immune response after concurrent topical application of nanoparticles and allergen. However, when present in allergen-adsorbed agglomerates, silica nanoparticles led to a low IgG/IgE ratio, a key risk factor of human atopic allergies. We suggest that minimizing interactions between nanomaterials and allergens will increase the safety of nanomaterials applied to skin.

**Electronic supplementary material:**

The online version of this article (doi:10.1186/s12989-015-0095-3) contains supplementary material, which is available to authorized users.

## Introduction

Because of their unique features and physicochemical properties [1, 2], nanomaterials are increasingly being used to add value to new and existing goods, such as cosmetics, foods, medicines, and industrial products [3–5]. However, these same novel features of nanomaterials make them hazardous under some conditions [6, 7]. To take full advantage of the potential benefits of nanomaterials, we must learn more about their hazards so that safer nanomaterials can be designed.

Numerous epidemiologic studies have prompted concerns regarding the health risks associated with exposure to nanomaterials; in particular, such studies have revealed that exposure to particulate matter (PM), including PM2.5 and Asian dust, induces many adverse effects, including facilitating the onset and severity of allergic diseases [8–11]. Because inhalational exposure to PM has been considered to be the main inducer of these adverse effects, this route has received the most attention regarding exposure to nanomaterials [12]. However, the skin is a major route of both intentional (from clothing, cosmetics, and other skin care products) and unintentional (from the environment) exposure to nanomaterials [13–15]. Furthermore, exposure to nanomaterials often occurs simultaneously with exposure to other chemical compounds and environmental allergens [16], but little is known about the hazards of cutaneous exposure to nanomaterials, particularly in combination with other substances.

In the current study, we investigated the effects of concurrent topical application of mite extract and amorphous silica nanoparticles, one of the most widely used nanomaterials, on allergic sensitization and AD in NC/Nga mice, a murine model of AD. We found that cutaneous exposure to the allergen and silica nanoparticles simultaneously did not aggravate AD-like skin lesions in the mice but resulted in low-level IgG production with little change in IgE production (IgE-biased immune response) and increased sensitivity to anaphylaxis. We suggest that an IgE-biased immune response was induced independently of the innate biologic effects of silica nanoparticles. Because a low IgG/IgE ratio is a characteristic feature of human atopic allergy, we believe that minimizing interactions between nanomaterials and allergens may improve the safety of cutaneously applied nanomaterials.

## Results and discussion

### Effects of co-exposure to mite allergen and silica nanoparticles in a murine model of AD

**Fig. 1:**
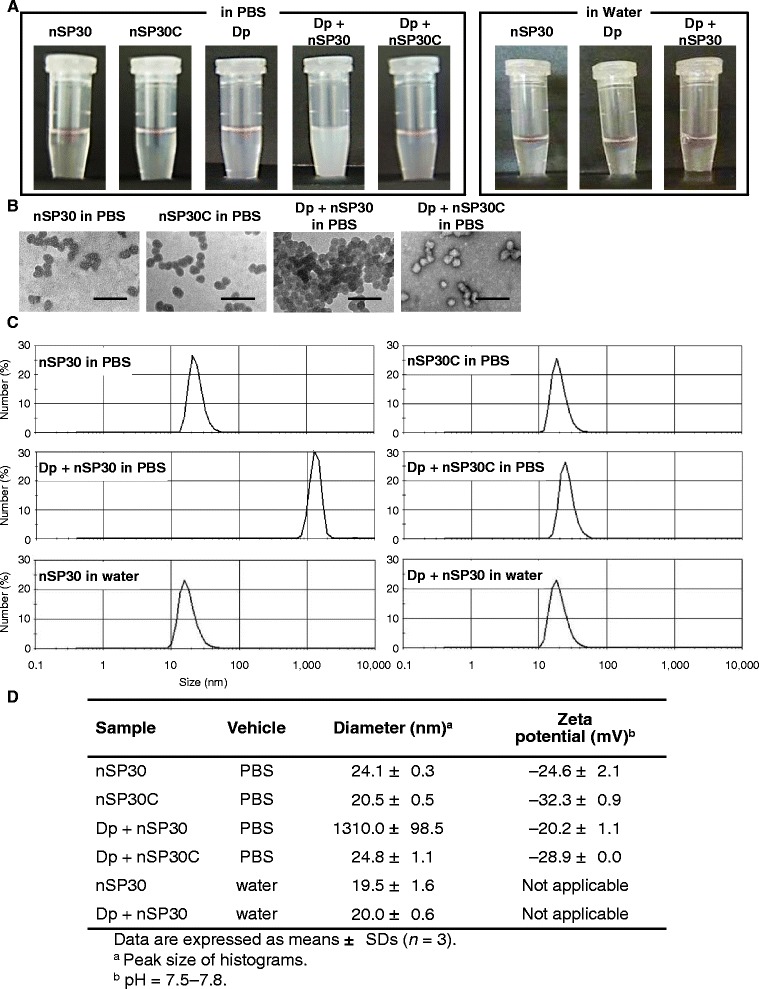
Physicochemical properties of silica nanoparticles alone and combined with allergen. **a** Macroscopic and (**b**) transmission electomicrographic images of the samples used in this study (scale bar, 100 nm). **c** Particle size distributions of samples diluted in PBS or water measured by using a dynamic light scattering method. **d** Mean particle diameters and zeta potentials of samples

For these experiments, we used silica nanoparticles with a diameter of 30 nm (nSP30). Solutions of nSP30 were clear and colorless (Fig. 1a), and transmission electron microscopy (TEM) revealed that the particles were smooth spheres (Fig. 1b). The size distribution spectrum of nSP30 was a single peak (Fig. 1c), and the mean hydrodynamic diameter (24.1 nm, as measured by means of a dynamic light-scattering method; Fig. 1d) corresponded almost exactly to the primary particle size. These results indicate that the nSP30 particles were well dispersed in solution.

**Fig. 2:**
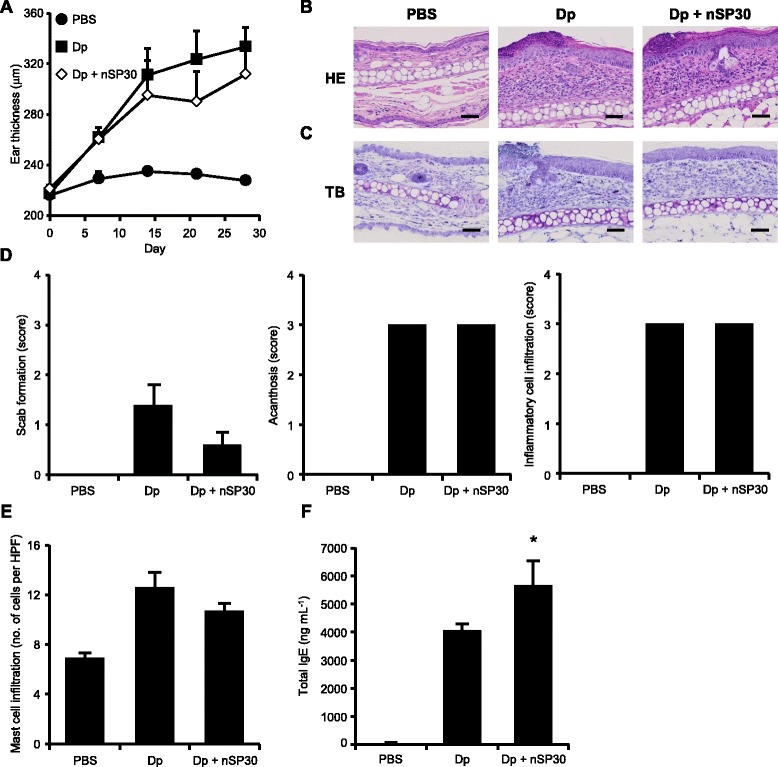
Induction of AD-like skin lesions by Dp + nSP30 agglomerates in PBS. **a** Effect of topical administration of Dp alone or Dp + nSP30 in PBS on ear thickness in NC/Nga mice. b and c, Histology of ear sections stained with (**b**) hematoxylin and eosin (HE) or (**c**) toluidine blue (TB). Scale bar, 50 μm. (**d**) Scores for several symptoms characteristic of AD evaluated in HE-stained sections. **e** Mast cell infiltration evaluated in TB-stained sections as the number of mast cells per high-power (400×) field (HPF). **f** Total plasma IgE concentrations measured 24 h after the final skin painting. Data are presented as means ± SEMs (*n* = 5). *P < 0.05 vs. Dp-alone group

To examine the effects of co-exposure of skin to allergen and nSP30, we used an extract of the mite *Dermatophagoides pteronyssinus* (Dp) and NC/Nga mice as a model for human AD [17]. Dp is a frequent cause of many allergic conditions, including asthma and AD [18, 19]. In addition, NC/Nga mice have a genetic skin barrier defect related to low ceramide production [20]. To induce AD-like skin lesions, we repeatedly cutaneously exposed NC/Nga mice to either Dp alone or a mixture of Dp and nSP30 in an isotonic solution (phosphate buffered saline; PBS). Note that although the solutions of Dp alone and nSP30 alone were clear and colorless, the mixture of Dp + nSP30 was cloudy (Fig. 1a). TEM images suggested that mixing resulted in the formation of agglomerates (Fig. 1b), which was confirmed by the fact that the mean hydrodynamic diameter of the particles in the mixture was 1310.0 nm, which was larger than that of nSP30 alone (Fig. 1c and d). First, we confirmed that exposure to nSP30 alone did not induce the formation of topical skin lesions (Additional file 1). Comparison of the PBS and Dp-alone groups indicated that cutaneous exposure to Dp induced ear thickening, scab formation, acanthosis, inflammatory cell infiltration, and mast cell infiltration (Fig. 2a–e). The effects of cutaneous exposure to Dp + nSP30 did not differ from those of Dp alone, except that the extent of ear thickening was slightly less in the Dp + nSP30 group than in the Dp-alone group.

Total IgE levels in plasma, which are often elevated in AD and other allergic conditions [21], were measured 24 h after the final treatment. The total IgE level in the Dp-alone group was higher than that in the PBS group (Fig. 2f), and the total IgE level in the Dp + nSP30 group was slightly higher than that in the Dp-alone group (Fig. 2f). Because a Th2-mediated immune response including IgE production is unnecessary for the development of AD-like skin lesions in NC/Nga mice [22], the high total IgE level induced by cutaneous exposure to Dp + nSP30 likely did not exacerbate the Dp-induced AD-like skin lesions in NC/Nga mice.

### Effect of cutaneous exposure to Dp + nSP30 on cutaneous allergic sensitization

**Fig. 3:**
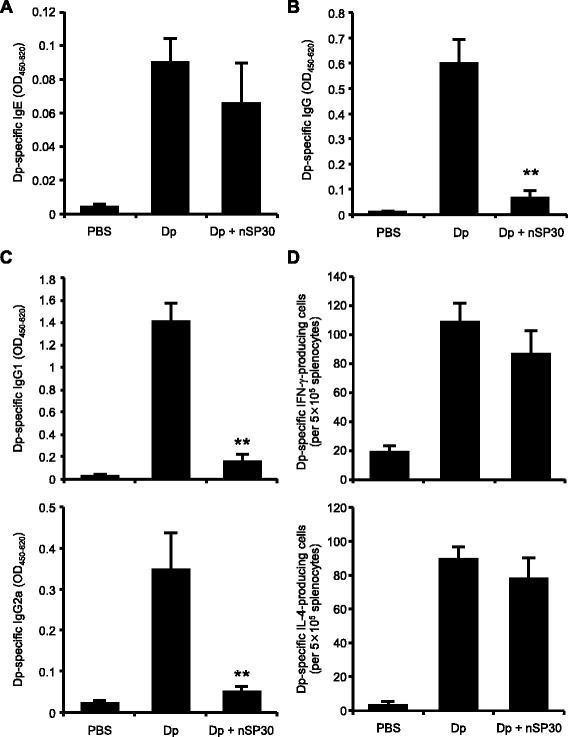
Induction of systemic immune responses by Dp + nSP30 agglomerates in PBS. **a–c** Plasma levels of Dp-specific (**a**) IgE, (**b**) IgG, and (**c**) IgG1 and IgG2a as analyzed by ELISA at 24 h after final topical treatment of NC/Nga mice with Dp alone or Dp + nSP30 in PBS. (D) Numbers of Dp-specific IFN-γ- and IL-4-producing splenocytes after re-stimulation with 100 μg mL^−1^ Dp, as determined by using ELISPOT assays specific for each cytokine. Data are given as means ± SEMs (*n* = 5–12). **P < 0.01 vs. Dp-alone group

To clarify the effect of topical Dp + nSP30 on cutaneous allergic sensitization, we evaluated the systemic immune responses 24 h after the final treatment. Although Dp-specific IgE levels were higher in both the Dp-alone group and the Dp + nSP30 group than in the PBS group, Dp-specific IgE levels did not differ significantly between the Dp-alone group and the Dp + nSP30 group (Fig. 3a). In contrast, the levels of Dp-specific total IgG and all evaluated IgG subtypes were significantly lower in the Dp + nSP30 group than in the Dp-alone group (Fig. 3b and c). In addition, we confirmed that nSP30 dose-dependently suppressed IgG production (Additional file 2). IgG subclass responses have been used to assess the type of immune response; IgG1 is known to indicate a Th2-type response, whereas IgG2a indicates a Th1 response [23]. Thus it is possible that skin exposure to Dp + nSP30 suppressed both the Th1 and the Th2 responses.

To further characterize the systemic immune responses, we enumerated the Dp-specific splenocytes secreting interferon-γ (IFN-γ) and interleukin-4 (IL-4) in each mouse by using cytokine-specific enzyme-linked immunosorbent spot (ELISPOT) assays (Fig. 3d). The numbers of Dp-specific IFN-γ- and IL-4-secreting splenocytes did not differ between the Dp-alone group and the Dp + nSP30 group. In addition, although concentration of IL-21 in the supernatants of splenocytes was significantly lower in the Dp + nSP30 group than in the Dp-alone group, none of the other measured cytokines (IL-5, 10, 13, and 17) differed between these groups (Additional file 3). Therefore, nSP30 might have affected IgG production without altering systemic Th1 and Th2 immune responses. Because IL-21 is a critical factor in IgG production [24, 25], additional study of IL-21 likely would help to clarify the mechanism underlying the reductions in both the IgG1 and IgG2a subtypes.

Recently, the skin was revealed to be the key initial site of allergic sensitization not only for allergic eczema but also for other atopic allergies, including allergic rhinitis, asthma, and food allergies [26–28]. Therefore, skin might play a special role in allergic sensitization. To this end, we assessed the effects of exposure to Dp + nSP30 by various other exposure routes on the IgG response (Additional file 4). Consistent with our previously reported results [29], nSP30-mediated IgG suppression was not observed after intranasal, oral, or intradermal administration of Dp + nSP30, thus suggesting that the low IgG response was a specific effect induced by cutaneous exposure to Dp + nSP30.

### Effect of cutaneous exposure to Dp + nSP30 on susceptibility to anaphylaxis

**Fig. 4:**
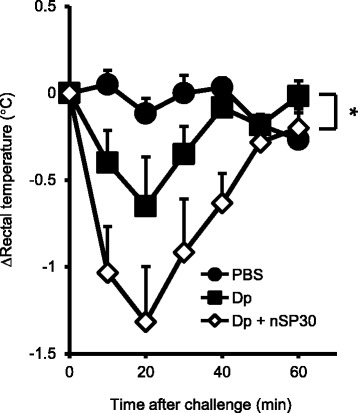
Sensitivity to anaphylactic shock. Time-dependence of change in rectal temperature in NC/Nga mice due to intravenous challenge with Dp after treatment with Dp alone or Dp + nSP30 in PBS. Data are given as means ± SEMs (*n* = 6). *P < 0.05 vs. Dp-alone group

Chemical mediators such as histamine and various cytokines induce the typical symptoms of atopic allergy, including inflammation and itching [30, 31]. The production of these chemical mediators is induced when an allergen cross-links IgE molecules bound to high-affinity IgE receptors (FcεRI) on mast cells and basophils. However, allergen-specific IgG, considered to be the ‘blocking antibody’, may neutralize allergen molecules before they can interact with IgE [32, 33]. Furthermore, IgG–allergen complexes act through a pathway regulated by the inhibitory receptor FcγRIIB to inhibit IgE-mediated mast cell and basophil signaling [32–34]. Therefore, the presence of allergen-specific IgG inhibits IgE-mediated allergic responses. Because our results showed that Dp + nSP30 led to low levels of Dp-specific IgG with little change in the levels of Dp-specific IgE (Fig. 3a–c), we examined whether this IgE-biased immune response induced by cutaneous exposure to Dp + nSP30 caused IgE-mediated hypersensitivity to Dp in a systemic anaphylaxis model. The decrease in rectal temperature after challenge with intravenous Dp was significantly greater in mice sensitized by Dp + nSP30 than in those sensitized by Dp alone (Fig. 4). That is, mice in the Dp + nSP30 group were more sensitive to the induction of Dp-specific anaphylaxis than were those in the Dp-alone group. Together, concurrent cutaneous exposure to Dp and nSP30 induced IgE-biased immune responses and, subsequently, increased sensitivity to anaphylaxis. In contrast, the functions of specific immunoglobulin subtypes vary between mice and humans; for example, blocking antibodies in humans are considered to be of the IgG4 subtype, which mice lack [35]. Therefore, our results cannot be extrapolated directly to humans. In addition, regulatory T and B cells have recently been suggested to be the main suppressors of atopic allergy in allergen-specific immunotherapy [36]. Additional study is needed to clarify whether increases in anaphylactic sensitivity is solely due to blocking antibody.

### Effects of sequential cutaneous exposure to allergen and nSP30 on the IgE-biased immune response

**Fig. 5:**
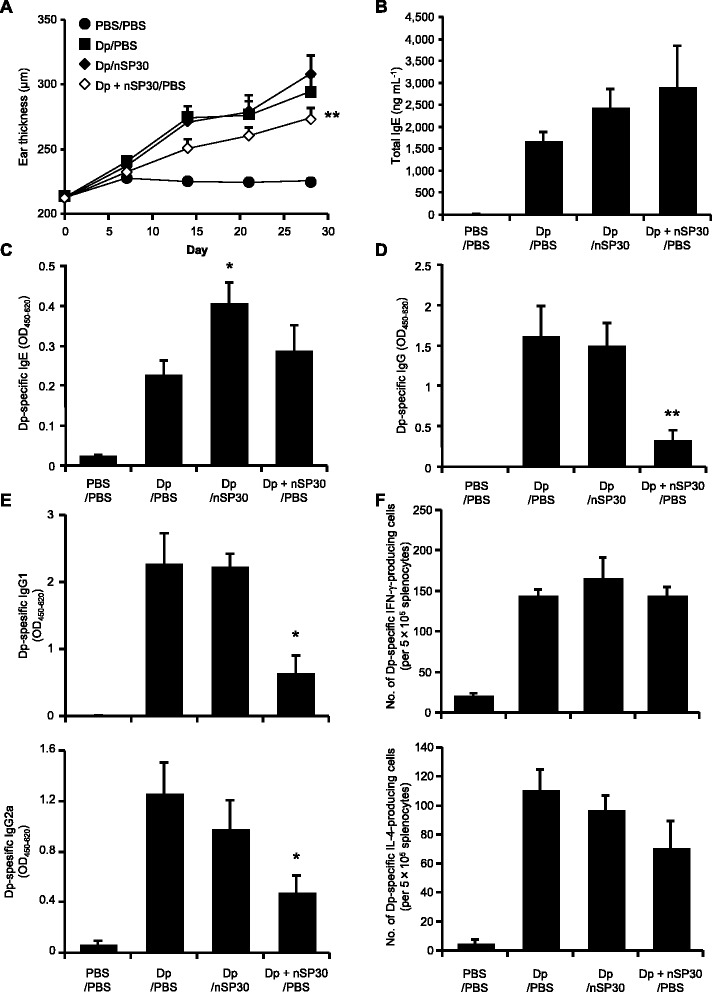
Effects of Dp + nSP30 aggregates compared with Dp and nSP30 administered on alternate days. **a** Effect of topical application of Dp + nSP30 and PBS (Dp + nSP30/PBS) compared with Dp and nSP30 (Dp/nSP30) on alternate days on ear thickness in NC/Nga mice. **b–e** Plasma levels of (**b**) total IgE, (**c**) Dp-specific IgE, (**d**) IgG and (**e**) IgG1 and IgG2a 24 h after final treatment, as analyzed by ELISA. **f** Numbers of Dp-specific IFN-γ- and IL-4-producing splenocytes after re-stimulation with 100 μg mL^−1^ Dp, as determined by ELISPOT assays specific for each cytokine. Data are presented as means ± SEMs (n = 5). *P < 0.05, **P < 0.01 vs. Dp/PBS group

We also evaluated the effects of sequential (rather than concurrent) cutaneous exposure to Dp and nSP30; that is, we topically applied Dp and nSP30 on alternate days rather than applying them together on the same day. Ear thickening and changes in total IgE concentration resulting from alternate-day application of Dp and nSP30 (Dp/nSP30) did not differ significantly from those resulting from concurrent treatment with Dp and PBS every other day (Dp/PBS) (Fig. 5a and b). Taken together, our results indicate that cutaneous exposure to nSP30 did not aggravate Dp-induced AD-like skin lesions regardless of whether the nanoparticles were applied together with or separately from Dp. In addition, alternate-day skin painting with Dp and nSP30 induced Dp-specific IgE and IgG production and Dp-specific IFN-γ- and IL-4-secreting splenocytes in the same way as did topical application of Dp and PBS (Fig. 5c–f). Therefore, exposure to both Dp and nSP30 was necessary to induce an IgE-biased immune response. Given that Dp + nSP30 formed agglomerates (Fig. 1), an interaction between Dp and nSP30 may have contributed to the subsequent IgE-biased immune response.

### Effects of agglomeration of allergen and nSP30 on the IgE-biased immune response

**Fig. 6:**
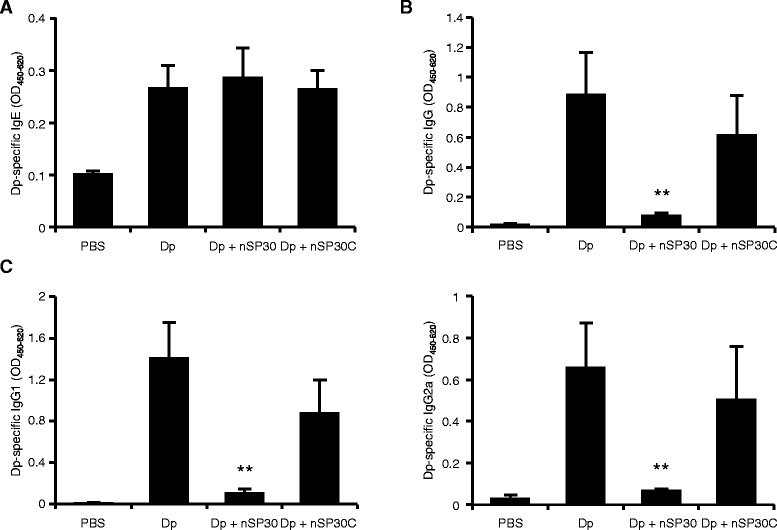
Prevention of Dp + nSP30-mediated IgE-biased immune response by surface modification of nSP30. **a**–**c** Plasma levels of Dp-specific (**a**) IgE, (**b**) IgG, and (**c**) IgG1 and IgG2a at 24 h after final treatment of NC/Nga mice with Dp alone, Dp + nSP30, or Dp + nSP30C, as analyzed by ELISA. Data are given as means ± SEMs (*n* = 5). *P < 0.05, **P < 0.01 vs. Dp-alone group

Because the surface properties of nanomaterials strongly influence their interactions with proteins [37], we investigated how surface modification of nSP30 with carboxyl groups affected the interaction between Dp and nSP30 and the subsequent IgE-biased immune response. Solutions of the mixture of Dp and nSP30 modified with surface carboxyl groups (nSP30C) were clear and colorless (Fig. 1a), and a TEM image of Dp + nSP30C was similar to that of nSP30C alone (Fig. 1b). The mean hydrodynamic diameter of Dp + nSP30C was 24.8 nm, which was only approximately 4 nm larger than that of nSP30C alone (Fig. 1c and d). These results suggest that the mixing of Dp and nSP30C did not result in substantial agglomeration. The level of Dp-specific IgE induced by cutaneous exposure to Dp + nSP30C was the same as that induced by cutaneous exposure to Dp alone or to Dp + nSP30 (Fig. 6a). Although cutaneous exposure to Dp + nSP30 significantly reduced the levels of Dp-specific IgG and its subtypes, cutaneous exposure to Dp + nSP30C had little effect on the levels of Dp-induced IgG, IgG1, and IgG2a (Fig. 6b and c).

**Fig. 7:**
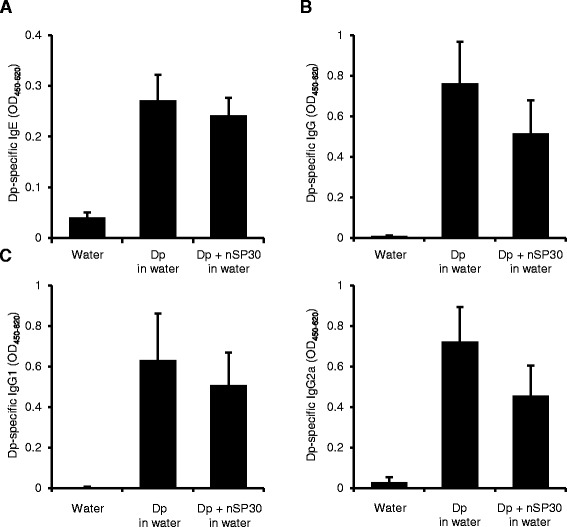
Effect of topical application of dispersed mixture of Dp + nSP30 on antibody response. a–c Plasma levels of Dp-specific (**a**) IgE, (**b**) IgG, and (**c**) IgG1 and IgG2a at 24 h after final topical treatment of NC/Nga mice with Dp alone or Dp + nSP30 in water, as analyzed by ELISA. Data are given as means ± SEMs (*n* = 5 or 6). *P < 0.05, **P < 0.01 vs. Dp-alone group

The vehicle in which nanoparticles are suspended is a key determinant of their dispersion and affects their toxicity [38, 39]. To further evaluate the effect of agglomeration of Dp and nSP30 on the IgE-biased immune response, we tested the effect of the vehicle on the formation of agglomerates and the IgE-biased immune response. Solutions of Dp and nSP30, either individually or together, in water were clear and colorless, but the combination of Dp and nSP30 in PBS was cloudy (Fig. 1a). The mean hydrodynamic diameter of nSP30 in water was 19.5 nm and that of Dp + nSP30 in water was 20.0 nm (Fig. 1c and d), indicating that mixing Dp and nSP30 in water did not cause agglomeration. We repeatedly painted NC/Nga mice with Dp + nSP30 in water and evaluated the Dp-induced antibody responses. When suspended in water, nSP30 had no effect on the level of Dp-specific IgE (Fig. 7a). In addition, the levels of Dp-specific total IgG, IgG1, and IgG2a did not differ between the Dp + nSP30 and Dp-alone groups when the vehicle used was water (Fig. 7b and c). Therefore the agglomeration of Dp and nSP30 might be necessary to impede the allergen-specific IgG response, but it did not affect the IgE response.

**Fig. 8:**
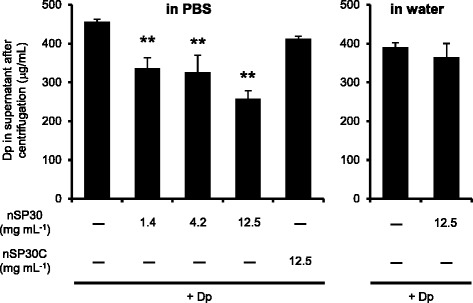
Evaluation of absorption of Dp to nSP30. The amount of Dp in the supernatant of each centrifuged sample was determined (OD_280_). Data are given as means ± SD (*n* = 3). **P < 0.01 vs. Dp-alone

Atopic patients typically have relatively low IgG/IgE ratios [40, 41]. In addition, human studies indicate that IgE production in the absence of IgG production is a key risk factor for the onset of atopic allergy [42]. Low-dose exposure to allergen sometimes induces a low IgG/IgE ratio in both mice and humans [43, 44]. Therefore, the formation of agglomerates in our experimental system may, in effect, decrease allergen doses and then induce an IgE-biased immune response. To assess the amount of unbound Dp, we compared the Dp concentration in the supernatant after centrifugation. After centrifugation, the Dp concentration in the supernatant of the Dp-alone group was somewhat lower than that observed before centrifugation (1000 μg/mL → 457 μg/mL) (Fig. 8). Mixing Dp and nSP30 together in PBS decreased the Dp concentrations in the supernatants in an nSP30-dependent manner (Fig. 8). In contrast, mixing Dp with nSP30C in PBS or with nSP30 in water had little effect on the Dp concentration in the supernatant compared with that of the Dp-alone group. Therefore, agglomeration might increase the amount of Dp absorbed to nSP30. Considering the fact that, in general, aggregates and agglomerates of nanomaterials have difficulty penetrating the skin barrier [45], the agglomeration of Dp and nSP30 might decrease the exposure dose of Dp somewhat even when the agglomerates release Dp on the skin.

We previously examined the Dp dose-effect in the same NC/Nga model (Hirai T. *et al.*, submitted paper). In that study, levels of both Dp-specific IgE and IgG were correlated with exposure amount of Dp in the range of 30 to 120 μg Dp mouse^−1^; in the current study, we used a dose of 120 μg Dp mouse^−1^ for the Dp-alone group. Furthermore, the Th2-related cytokine response in Dp-stimulated splenocytes was lower in the120-μg Dp mouse^−1^ group than in other (lower) dose groups. In contrast, we show here that the exposure to agglomerates of Dp and nSP30 in PBS induced a dampened Dp-specific IgG response with little change in the Dp-specific IgE and Th1/Th2 immune responses compared with those of the Dp-alone group (Fig. 3 and Additional file 3). Together, these observations suggest that the IgE-biased immune response in the current study was not solely due to the low Dp exposure resulting from the agglomeration of Dp and nSP30. Perhaps the agglomerates of Dp and nSP30 in PBS not only decreased the Dp exposure dose but also behaved as a ‘depot,’ thus controlling the allergen concentration, prolonging allergen exposure, and subsequently causing IgE-biased allergic sensitization. We believe additional studies that focus on the effect of nanoparticles on allergen penetration kinetics in the skin would be beneficial to confirm the safety of cutaneous exposure to nanomaterials. However, we acknowledge the need for additional studies in which the exposure scenario is more representative of that in humans to define the safety of concurrent cutaneous exposure to nanomaterials and allergen.

It is well recognized that there are some situations in our daily lives, the development of atopic allergy is inhibited by higher dose exposure to allergen due to high induction of blocking IgG [46–48]. Particularly in these high-dose exposure situations, we suggest that allergen-nanoparticle agglomerates, by inducing IgE-biased immune responses, might play a critical role in the development of atopic allergy. In addition, the IgE-biased immune response induced by cutaneous exposure to agglomerates of allergen and nanoparticles in our mice was similar to those humans with atopic allergies, who often have a low IgG/IgE ratio, as mentioned earlier [40, 41]. Therefore epidemiologic studies that address cutaneous as well as inhalational exposure to nanomaterials may improve our understanding of the onset of atopic allergy.

Recently, Ilves M. *et al.* described the effects of cutaneous exposure to nano-sized ZnO (nZnO) administered with model antigens, ovalbumin and staphylococcal enterotoxin B, on AD-like skin lesions and antibody responses [49]. Interestingly, the effects observed for nZnO and an antigen were similar to the effects of agglomerates of Dp and nSP30: nZnO suppressed allergen-induced skin inflammation and induced low-level IgG production in the context of a high IgE response. The authors of the previous study [49] did not address changes of nZnO dispersibility by mixing allergen, but considering that nZnO is predisposed to forming agglomerates and might adsorb a coexisting substance [50], nZnO might play similar role to that of nSP30. To better understand the hazards of nanomaterials so that we can maximize their potential benefits, we should pay increased attention to the state of nanoparticles (*e.g.* dispersibility) in administration in nanotoxicology studies. We consider this focus particularly applicable in the hazard evaluation of nanomaterials that are in the presence of other substances, which could interact with them.

Cutaneous exposure to aggregates or agglomerates of nanomaterials is generally considered to be safer than is similar exposure to individual nanoparticles, mainly because agglomerates of nanomaterials have difficulty penetrating the skin [50]. However, Dp and nSP30 induced an IgE-biased immune response only when they formed agglomerates. Although these results represent only indirect effects of nanomaterials, we think that hazard identification is necessary even when nanomaterials are considered to be unable to cross the skin barrier (*e.g.* when nanomaterials form aggregates or agglomerates). In contrast, surface modification of the nSP30 with carboxyl groups suppressed the adsorption of the allergen and did not induce IgE-biased allergic sensitization (Fig. 6). An increased understanding of the regulatory factors that induce the agglomeration of silica nanoparticles is crucial for appropriate regulation of the surface properties of nanomaterials so that they can be used safely.

## Conclusions

Cutaneous exposure to agglomerates of Dp and nSP30 induced an IgE-biased immune response in NC/Nga mice and increased their sensitivity to anaphylaxis. Surprisingly, these results were independent of the innate biologic effects of nSP30; these results required both simultaneous exposure to Dp and nSP30 and their agglomeration. In particular, features of the mice with IgE-biased immune response induced by cutaneous exposure to agglomerates of allergen and nanoparticles resembled those of humans with atopic allergies, who often have a low IgG/IgE ratio; follow-up epidemiologic studies that focus cutaneous compared with inhalational exposure to nanomaterials might improve our understanding of the onset of atopic allergy. In light of our findings, we suggest that minimizing the interaction between nanomaterials and allergens may improve the safety of topically applied products containing nanomaterials.

## Methods

### Silica nanoparticles

nSP30 and nSP30C (nSP30C is a surface modified nSP30 with carboxyl groups) silica nanoparticles (diameter, 30 mm) were purchased from Micromod Partikeltechnologie (Rostock/Warnemünde, Germany). Suspensions of silica nanoparticles were stored at room temperature. Immediately prior to use, the suspensions were sonicated at 400 W for 5 min at 25 °C and then vortexed for 1 min.

### Mice

Female NC/Nga slc mice were purchased from SLC (Kyoto, Japan) and used at 6 wk of age. All animal experiments were performed in accordance with the institutional guidelines of Osaka University and National Institute of Biomedical Innovation regarding the ethical treatment of animals.

### Preparation of the mixtures of Dp and nSP30s

To prepare the mixtures of nSP30s and Dp (Cosmo Bio LSL, Tokyo, Japan), we first combined nSP30s (25 mg mL^−1^ in water) and concentrated PBS (1.1× to 4.6×; the concentration of the PBS stock used depended on the concentration of nSP30s needed in the final sample, the diluent for which was 1× PBS). We then added the stock solution of Dp (2.78 mg protein mL^−1^ in PBS) to the solutions containing various concentrations of nSP30s. As soon as the nSP30s and Dp solutions were combined, the mixtures were vortexed for 1 min, allowed to incubate at room temperature for 0.5 to 1.5 h, and vortexed again for 1 min just prior to use.

### Physicochemical examination of silica nanoparticles

TEM (H-7650; Hitachi High-Technologies Corporation, Tokyo, Japan) was used to assess the size and shape of the silica nanoparticles. nSP30 and nSP30C nanoparticles were diluted to 0.25 mg mL^−1^ in PBS or deionized water, and the mean particle diameter and zeta potential at 25 °C were measured by using a Zetasizer Nano ZS (Malvern Instruments, Worcestershire, UK). Specifically, the mean diameters and particle size distributions of the nanoparticles were measured by means of a dynamic light-scattering method, whereas zeta potentials were measured by laser Doppler electrophoresis; both types of measurements were performed by using capillary cells (Malvern Instruments). The pH of each particle suspension was measured by using an ISFET pH meter (Shindengen, Tokyo, Japan).

### Skin painting and assessment of allergic response

Using a hair-removal cream (Epilat; Kracie, Tokyo, Japan), we removed the hair from the backs of all mice twice during each experiment (on day 1 and on day 18–20). Mice were treated either with Dp (1 mg mL^−1^ fin. conc) alone or with silica nanoparticles (nSP30 or nSP30C, 12.5 mg mL^−1^ fin. conc.) in 120 μL PBS by painting the ventral side of both ears and the depiliated dorsum (20 μL per ear and 80 μL on the back) on alternate days or every third day for 4 wk for a total of 13 applications. Additional mice were painted with Dp only on one day followed by painting with nSP30 alone on the next day; this alternating pattern was repeated for a total of 13 applications of each solution over 4 wk. Regardless of the dose schedule used, any sample remaining from a previous application was removed by gently wiping with a disposable wipe (Kim wipes, Crecia, Tokyo, Japan) wetted with 70 % ethanol.

We used a dial thickness gauge (0.001-mm type; Ozaki Manufacturing, Tokyo, Japan) to measure ear thickness weekly. To evaluate the severity of the IgE-mediated allergic response’ instead, we used a systemic anaphylaxis model. Specifically, at 1 wk after the final skin painting, each mouse was challenged with an intravenous injection of Dp (15 μg in 200 μL PBS). The severity of anaphylactic shock was assessed according to the change in rectal temperature measured by using a digital thermometer (KN-91; Natsume Seisakusho, Tokyo, Japan).

### Histologic analysis

At 24 h after the final topical treatment, the ears of euthanized mice were removed, placed in fixative solution (4 % paraformaldehyde in PBS; Wako, Osaka, Japan), embedded in paraffin, and sectioned. The tissue sections were stained with hematoxylin and eosin or with toluidine blue. Histopathological examination was performed by the Applied Medical Research Laboratory (Osaka, Japan). For each sample, representative symptoms of AD (scab formation, acanthosis and inflammatory cell infiltration) were scored as follows: 0, none; 1, slight; 2, mild; 3, moderate; and 4, severe. In addition, we counted the number of mast cells in 3 random high-power fields (magnification, 400×).

### Blood sampling

Using hematocrit capillary tubes (Terumo, Tokyo, Japan), we obtained blood samples from the retro-orbital venous plexus once each week during the study period. At 24 h after the final skin painting, blood was collected by cardiocentesis into heparin-treated syringes and centrifuged at 3000 × *g* at 4 °C; the resulting plasma was stored at −80 °C until analysis.

### Quantitation of total IgE concentration

The total IgE concentration in plasma was measured by using an ELISA kit (BD Biosciences, San Diego, CA, USA) according to the manufacturer’s instructions.

### Detection of Dp-specific antibody

The levels of Dp-specific antibody in plasma were determined by ELISA. To detect IgG, IgG1, IgG2a, IgG2b and IgG3, we coated ELISA plates (Maxisorp, type 96 F; Nunc A/S, Roskilde, Denmark) with Dp in PBS (50 μg mL^−1^). The coated plates were incubated with 2 % Block Ace (Dainippon Sumitomo Pharmaceuticals, Osaka, Japan). Plasma samples were diluted by 2 % Block Ace and these dilutions were added to the Dp-coated plates. After incubation with plasma, the coated plates were incubated with a horseradish peroxidase–conjugated goat anti-mouse IgG, IgG1, IgG2a, IgG2b or IgG3 solution (SouthernBiotech, Birmingham, AL, USA) for two hours at room temperature. After the incubation, the color reaction was developed with tetramethyl benzidine (Moss, Inc.; Pasadena, MD, USA), stopped with 2 N H_2_SO_4_, and measured at OD_450–620_ on a microplate reader.

To detect Dp-specific IgE, we coated ELISA plates (Maxisorp, type 96 F) with purified rat anti-mouse IgE (2 μg mL^−1^; R35-72, BD Biosciences;), incubated them with Block Ace, and added samples of diluted plasma as described earlier. Treated plates were incubated with biotin-conjugated Dp (5 μg mL^−1^) followed by horseradish peroxidase–coupled streptavidin (Southern Biotechnology Associates). Color detection was performed as described earlier.

### Isolation of splenocytes

Spleens were removed aseptically and placed in RPMI 1640 (Wako) supplemented with 10 % fetal bovine serum, 10 mL L^−1^ of a 100× nonessential amino acid solution (Gibco, Invitrogen, Carlsbad, CA, USA), 50 μM 2-mercaptoethanol (Gibco), and 1 % antibiotic cocktail (10,000 U mL^−1^ penicillin, 10,000 μg mL^−1^ streptomycin, 25 μg mL^−1^ amphotericin B; Gibco). Single-cell suspensions of splenocytes were treated with ammonium chloride to lyse the red blood cells; treated splenocytes then were washed, counted, and suspended in RPMI 1640.

### Cytokine assays

To determine antigen-specific cytokine (IFN-γ and IL-4) responses, splenocytes (5 × 10^5^ cells well^−1^) were stimulated with Dp antigen (100 μg mL^−1^) *in vitro*. After 24 h incubation at 37 °C (95 % room air, 5 % CO2), stimulated splenocytes were washed, and the numbers of IFN-γ- and IL-4-producing cells were determined by using an ELISPOT assay kit (BD Biosciences) according to the manufacturer’s instructions.

### Measurement of amount of Dp adsorbed to silica nanoparticles

Solutions of Dp (1 mg mL^−1^ fin. conc.) + each nSP immediately after preparation in PBS or water for 30 min at 25 °C were centrifuged (2 h, 40,000 × *g*, 4 °C). The amount of Dp in the supernatant then was determined spectrophotometrically (OD_280_; Nanodrop 2000, Thermo Scientific, Kanagawa, Japan).

### Statistical analysis

Statistical analyses were performed with Ekuseru-Toukei 2010 software (Social Survey Research Information Co., Ltd., Tokyo, Japan). All data are presented as means ± SEMs. Significant differences between control groups and experimental groups were determined by using the Dunnett test; a *P* value less than 0.05 was considered significant.

Methods for additional files are available in (Additional file 5).

## Additional files

Additional file 1:
**Effects of nSP30 nanoparticles alone on skin.** (A) Effect of topical treatment with nSP30 alone on ear thickness in NC/Nga mice. (B, C) Histology of ear sections stained with (B) hematoxylin and eosin (HE) or (C) toluidine blue (TB). Scale bar, 50 μm. (D) Scores for several symptoms characteristics of AD as evaluated in HE-stained sections. (E) Mast cell infiltration evaluated in TB-stained as the number of mast cells per high-power (*i.e.*, 400×) field. (F) Total IgE concentration at 24 h after the final treatment. N. D., not detected (<30 ng mL-1). Data are given as mean ± SEMs (n = 5). Additional file 2:
**Dose-dependence of the effects of nSP30 nanoparticles on mite-allergen (Dp)-specific antibody responses.** A-C, Plasma levels of Dp-specific (A) IgE, (B) IgG, and (C) IgG subtypes in plasma collected from NC/Nga mice after topical treatment with Dp alone or with 1.4, 4.2, or 12.5 mg mL-1 nSP30 (as analyzed by ELISA). Data are given as mean ± SEMs (n = 5). **P* < 0.05. ***P* < 0.01 vs. Dp-alone group. Additional file 3:
**Effects of nSP30 nanoparticles on effector cytokines induced by mite allergen (Dp).** Levels of Dp-specific effector cytokines in the supernatants of NC/Nga mouse splenocytes stimulated with 100 μg mL-1 Dp for 4 days (determined by use of cytokines immunoassay or ELISA). Data are given as means ± SEMs (n = 5). **P* < 0.05. ***P* < 0.01 vs. Dp-alone group. Additional file 4:
**Effects of administration route of mite allergen (Dp) + nSP30 mamoparticles on Dp-sepcific antibody response.** Levels of Dp-specific IgE and IgG in plasma collected from NC/Nga mice after treatment with Dp alone or Dp + nSP30 by intradermal (foot-pad injection), intranasal, or oral administration (as analyzed by ELISA). Data are given as means ± SEMs (n = 3-6). ***P* < 0.01 vs. Dp-alone group. Additional file 5:
**Additional methods.**
