# Supplementary material for: Cutaneous exposure to agglomerates of silica nanoparticles and allergen results in IgE-biased immune response and increased sensitivity to anaphylaxis in mice
Source: Part Fibre Toxicol. 2015 Jun 26;12:16. doi: 10.1186/s12989-015-0095-3 (PMC4482284; doi:10.1186/s12989-015-0095-3)
Supplement: Additional file 1: — Effects of nSP30 nanoparticles alone on skin. (A) Effect of topical treatment with nSP30 alone on ear thickness in NC/Nga mice. (B, C) Histology of ear sections stained with (B) hematoxylin and eosin (HE) or (C) toluidine blue (TB). Scale bar, 50 μm. (D) Scores for several symptoms characteristics of AD as evaluated in HE-stained sections. (E) Mast cell infiltration evaluated in TB-stained as the number of mast cells per high-power (i.e., 400×) field. (F) Total IgE concentration at 24 h after the final treatment. N. D., not detected (<30 ng mL-1). Data are given as mean ± SEMs (n = 5). [file 12989_2015_95_MOESM1_ESM.pptx]

## Slide 1
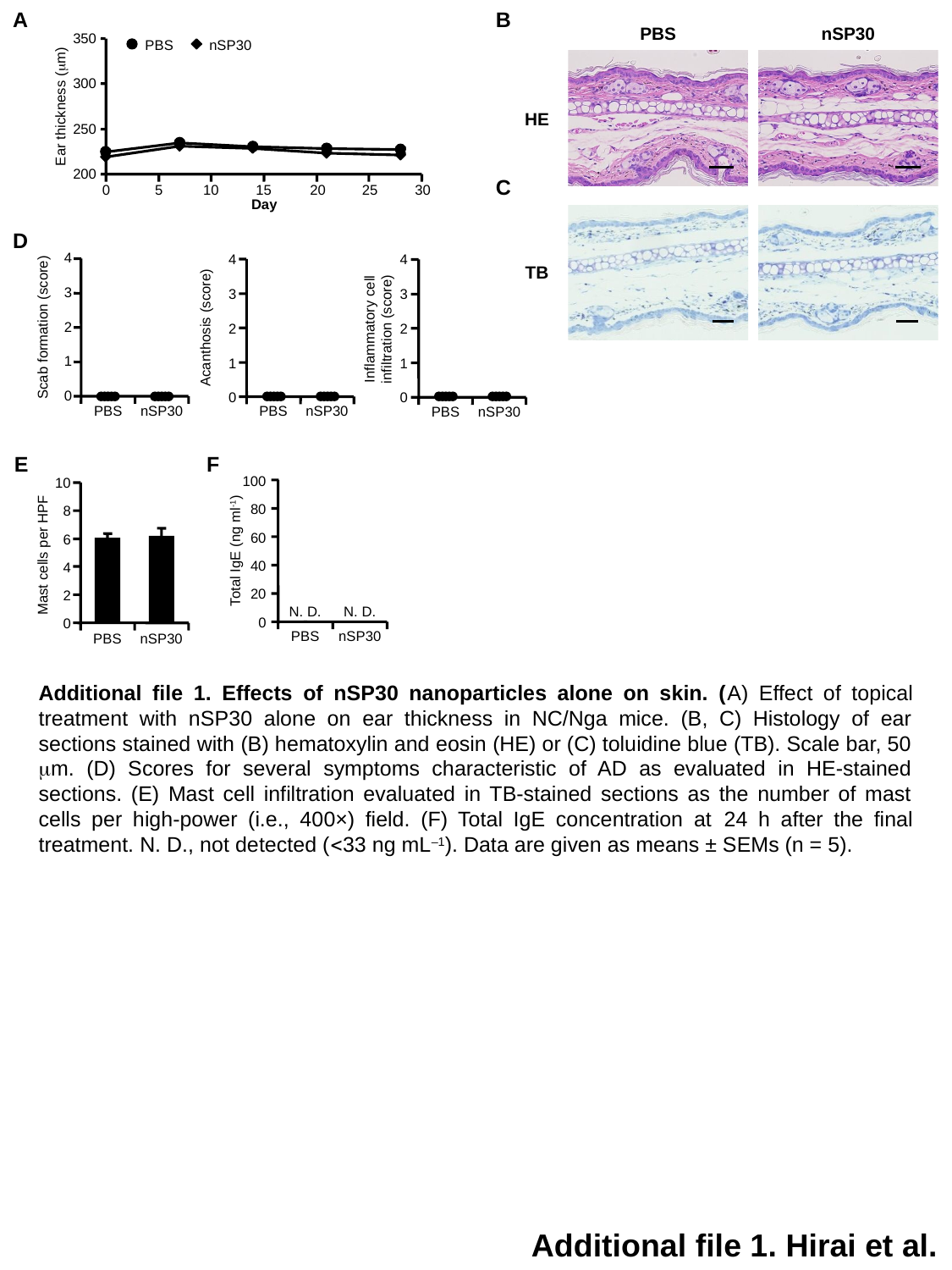

A
B
PBS
nSP30
350
PBS
nSP30
300
Ear thickness (μm)
HE
250
200
C
0
5
10
15
20
25
30
Day
D
4
4
4
TB
3
3
3
Inflammatory cell infiltration (score)
2
Scab formation (score)
Acanthosis (score)
2
2
1
1
1
0
0
0
PBS
nSP30
PBS
nSP30
PBS
nSP30
E
F
100
10
80
8
nSP30
60
6
PBS
Total IgE (ng ml-1)
Mast cells per HPF
40
4
20
2
N. D.
N. D.
0
0
PBS
nSP30
Additional file 1. Effects of nSP30 nanoparticles alone on skin. (A) Effect of topical treatment with nSP30 alone on ear thickness in NC/Nga mice. (B, C) Histology of ear sections stained with (B) hematoxylin and eosin (HE) or (C) toluidine blue (TB). Scale bar, 50 μm. (D) Scores for several symptoms characteristic of AD as evaluated in HE-stained sections. (E) Mast cell infiltration evaluated in TB-stained sections as the number of mast cells per high-power (i.e., 400×) field. (F) Total IgE concentration at 24 h after the final treatment. N. D., not detected (33 ng mL–1). Data are given as means ± SEMs (n = 5).
Additional file 1. Hirai et al.
