# Supplementary material for: Cutaneous exposure to agglomerates of silica nanoparticles and allergen results in IgE-biased immune response and increased sensitivity to anaphylaxis in mice
Source: Part Fibre Toxicol. 2015 Jun 26;12:16. doi: 10.1186/s12989-015-0095-3 (PMC4482284; doi:10.1186/s12989-015-0095-3)
Supplement: Additional file 2: — Dose-dependence of the effects of nSP30 nanoparticles on mite-allergen (Dp)-specific antibody responses. A-C, Plasma levels of Dp-specific (A) IgE, (B) IgG, and (C) IgG subtypes in plasma collected from NC/Nga mice after topical treatment with Dp alone or with 1.4, 4.2, or 12.5 mg mL-1 nSP30 (as analyzed by ELISA). Data are given as mean ± SEMs (n = 5). *P < 0.05. **P < 0.01 vs. Dp-alone group. [file 12989_2015_95_MOESM2_ESM.pptx]

## Slide 1
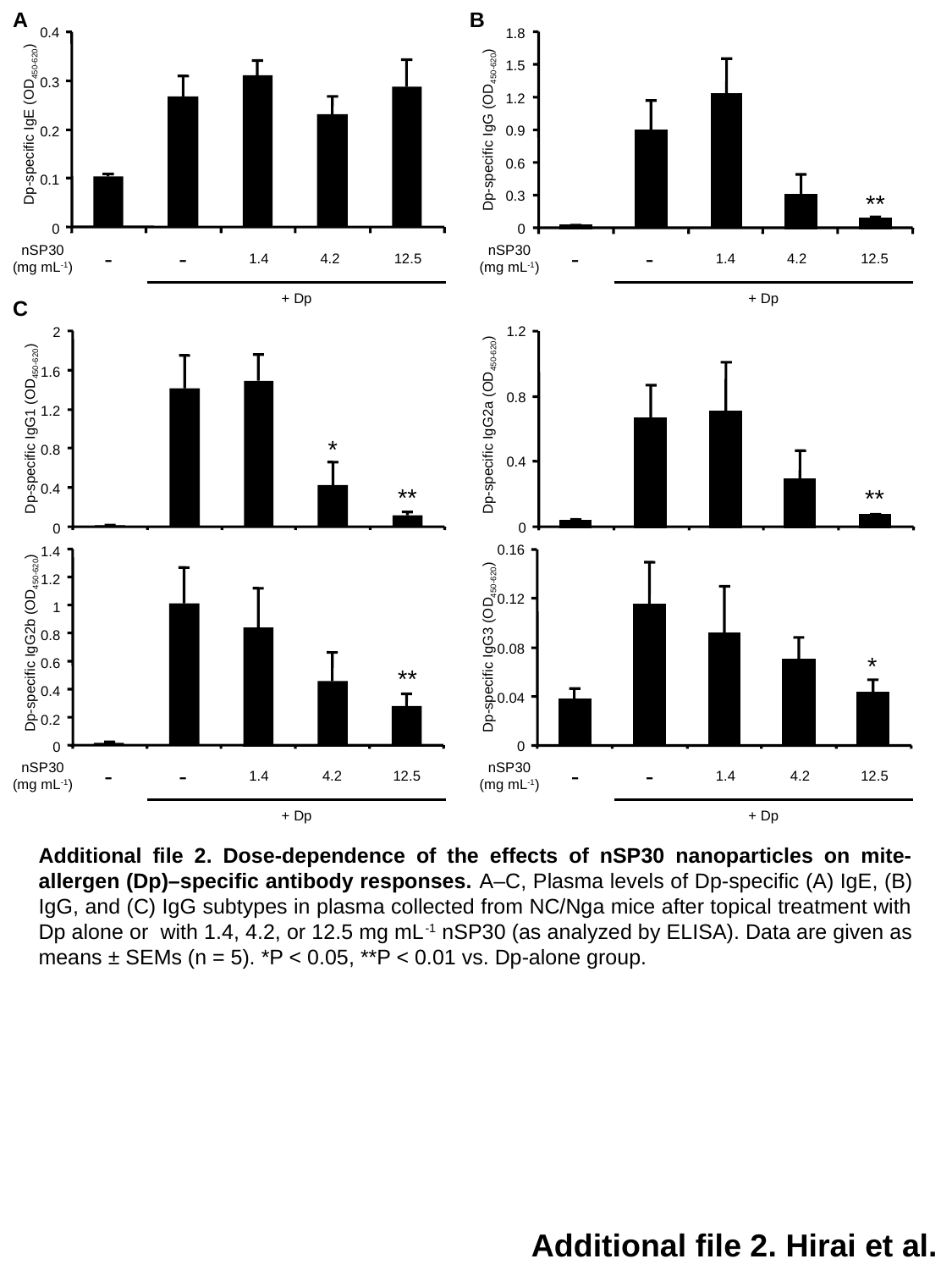

A
B
0.4
0.3
Dp-specific IgE (OD450-620)
0.2
0.1
0
1.8
1.5
1.2
Dp-specific IgG (OD450-620)
0.9
0.6
**
0.3
0
nSP30
(mg mL-1)
-
-
1.4
4.2
12.5
+ Dp
nSP30
(mg mL-1)
-
-
1.4
4.2
12.5
+ Dp
C
1.2
2
1.6
0.8
1.2
Dp-specific IgG2a (OD450-620)
Dp-specific IgG1 (OD450-620)
*
0.8
0.4
**
**
0.4
0
0
0.16
1.4
1.2
0.12
1
0.8
Dp-specific IgG2b (OD450-620)
Dp-specific IgG3 (OD450-620)
0.08
*
0.6
**
0.4
0.04
0.2
0
0
nSP30
(mg mL-1)
-
-
1.4
4.2
12.5
+ Dp
nSP30
(mg mL-1)
-
-
1.4
4.2
12.5
+ Dp
Additional file 2. Dose-dependence of the effects of nSP30 nanoparticles on mite-allergen (Dp)–specific antibody responses. A–C, Plasma levels of Dp-specific (A) IgE, (B) IgG, and (C) IgG subtypes in plasma collected from NC/Nga mice after topical treatment with Dp alone or with 1.4, 4.2, or 12.5 mg mL-1 nSP30 (as analyzed by ELISA). Data are given as means ± SEMs (n = 5). *P < 0.05, **P < 0.01 vs. Dp-alone group.
Additional file 2. Hirai et al.
