# Supplementary material for: Cutaneous exposure to agglomerates of silica nanoparticles and allergen results in IgE-biased immune response and increased sensitivity to anaphylaxis in mice
Source: Part Fibre Toxicol. 2015 Jun 26;12:16. doi: 10.1186/s12989-015-0095-3 (PMC4482284; doi:10.1186/s12989-015-0095-3)
Supplement: Additional file 3: — Effects of nSP30 nanoparticles on effector cytokines induced by mite allergen (Dp). Levels of Dp-specific effector cytokines in the supernatants of NC/Nga mouse splenocytes stimulated with 100 μg mL-1 Dp for 4 days (determined by use of cytokines immunoassay or ELISA). Data are given as means ± SEMs (n = 5). *P < 0.05. **P < 0.01 vs. Dp-alone group. [file 12989_2015_95_MOESM3_ESM.pptx]

## Slide 1
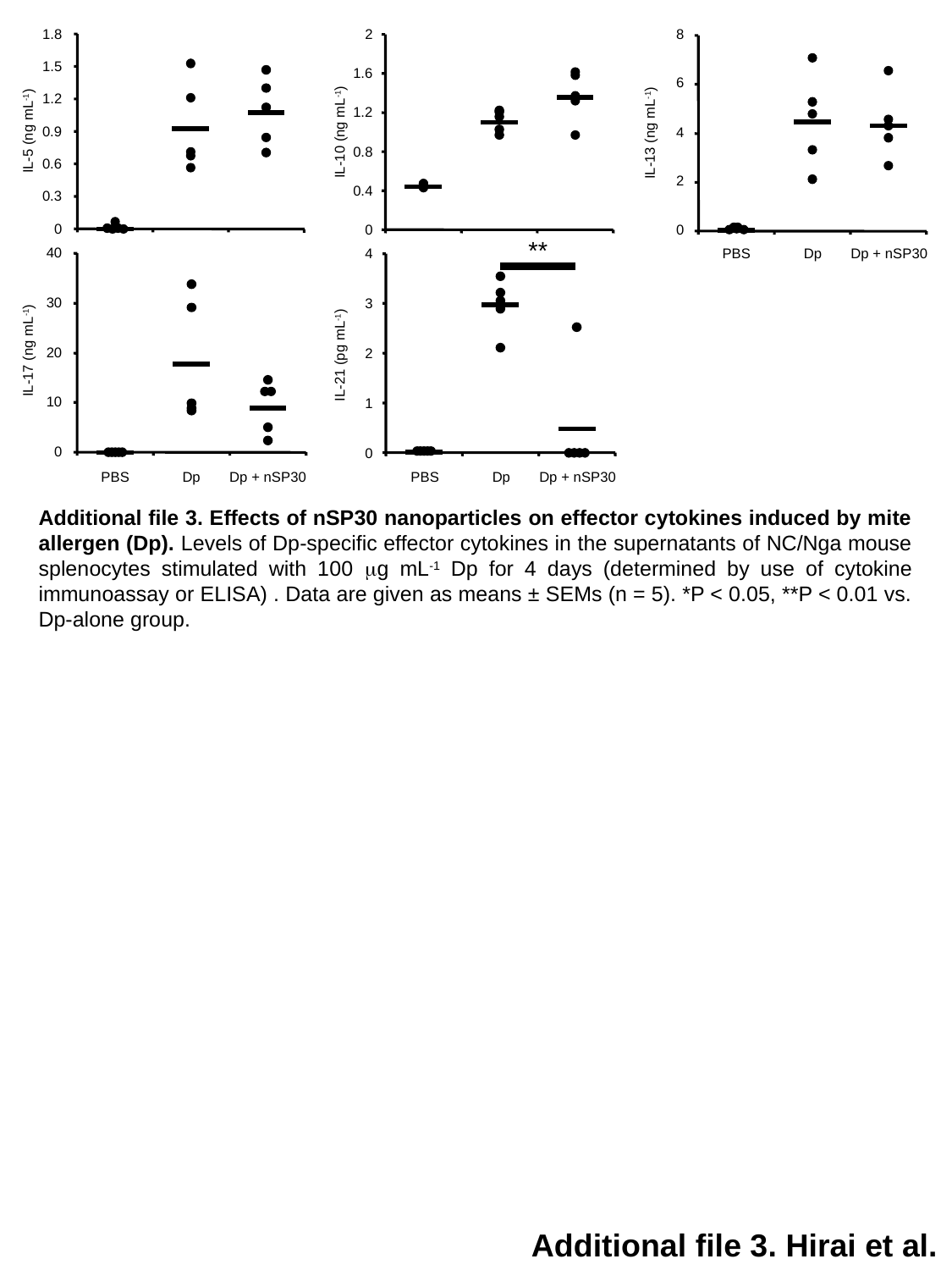

1.8
8
2
1.5
1.6
6
1.2
1.2
0.9
IL-5 (ng mL-1)
4
IL-10 (ng mL-1)
IL-13 (ng mL-1)
0.8
0.6
2
0.4
0.3
0
0
0
**
40
4
PBS
Dp
Dp + nSP30
30
3
IL-17 (ng mL-1)
20
2
IL-21 (pg mL-1)
10
1
0
0
PBS
Dp
Dp + nSP30
PBS
Dp
Dp + nSP30
Additional file 3. Effects of nSP30 nanoparticles on effector cytokines induced by mite allergen (Dp). Levels of Dp-specific effector cytokines in the supernatants of NC/Nga mouse splenocytes stimulated with 100 μg mL-1 Dp for 4 days (determined by use of cytokine immunoassay or ELISA) . Data are given as means ± SEMs (n = 5). *P < 0.05, **P < 0.01 vs. Dp-alone group.
Additional file 3. Hirai et al.
