# Supplementary material for: Cutaneous exposure to agglomerates of silica nanoparticles and allergen results in IgE-biased immune response and increased sensitivity to anaphylaxis in mice
Source: Part Fibre Toxicol. 2015 Jun 26;12:16. doi: 10.1186/s12989-015-0095-3 (PMC4482284; doi:10.1186/s12989-015-0095-3)
Supplement: Additional file 4: — Effects of administration route of mite allergen (Dp) + nSP30 mamoparticles on Dp-sepcific antibody response. Levels of Dp-specific IgE and IgG in plasma collected from NC/Nga mice after treatment with Dp alone or Dp + nSP30 by intradermal (foot-pad injection), intranasal, or oral administration (as analyzed by ELISA). Data are given as means ± SEMs (n = 3-6). **P < 0.01 vs. Dp-alone group. [file 12989_2015_95_MOESM4_ESM.pptx]

## Slide 1
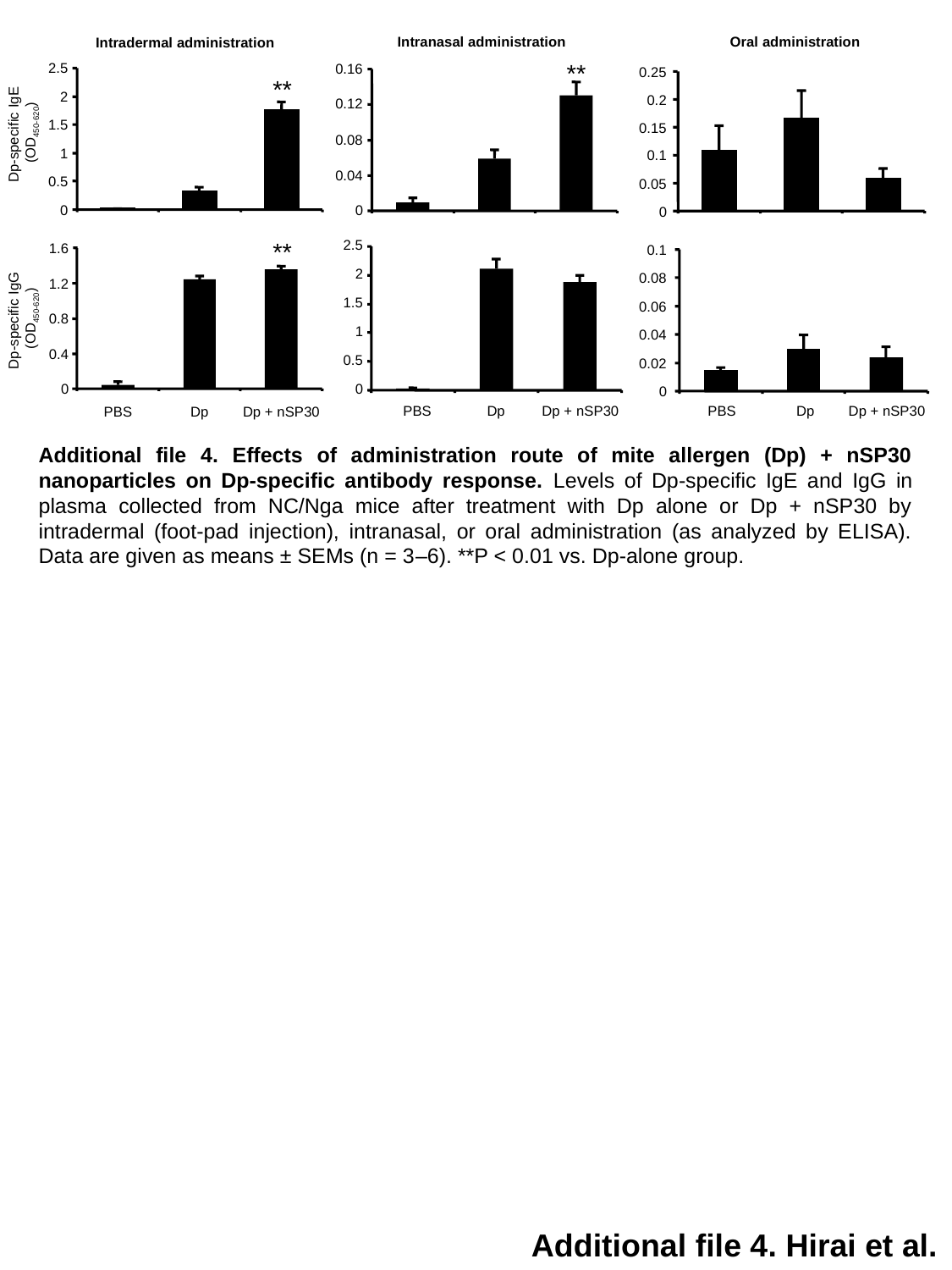

Intranasal administration
Oral administration
Intradermal administration
**
2.5
0.16
0.12
0.08
0.04
0
0.25
**
2
0.2
Dp-specific IgE
(OD450-620)
1.5
0.15
1
0.1
0.5
0.05
0
0
**
2.5
1.6
0.1
2
0.08
1.2
1.5
0.06
Dp-specific IgG
(OD450-620)
0.8
1
0.04
0.4
0.5
0.02
0
0
0
PBS
Dp
Dp + nSP30
PBS
Dp
Dp + nSP30
PBS
Dp
Dp + nSP30
Additional file 4. Effects of administration route of mite allergen (Dp) + nSP30 nanoparticles on Dp-specific antibody response. Levels of Dp-specific IgE and IgG in plasma collected from NC/Nga mice after treatment with Dp alone or Dp + nSP30 by intradermal (foot-pad injection), intranasal, or oral administration (as analyzed by ELISA). Data are given as means ± SEMs (n = 3–6). **P < 0.01 vs. Dp-alone group.
Additional file 4. Hirai et al.
