# Supplementary material for: Cutaneous exposure to agglomerates of silica nanoparticles and allergen results in IgE-biased immune response and increased sensitivity to anaphylaxis in mice
Source: Part Fibre Toxicol. 2015 Jun 26;12:16. doi: 10.1186/s12989-015-0095-3 (PMC4482284; doi:10.1186/s12989-015-0095-3)
Supplement: Additional file 5: — Additional methods. [file 12989_2015_95_MOESM5_ESM.pptx]

## Slide 1
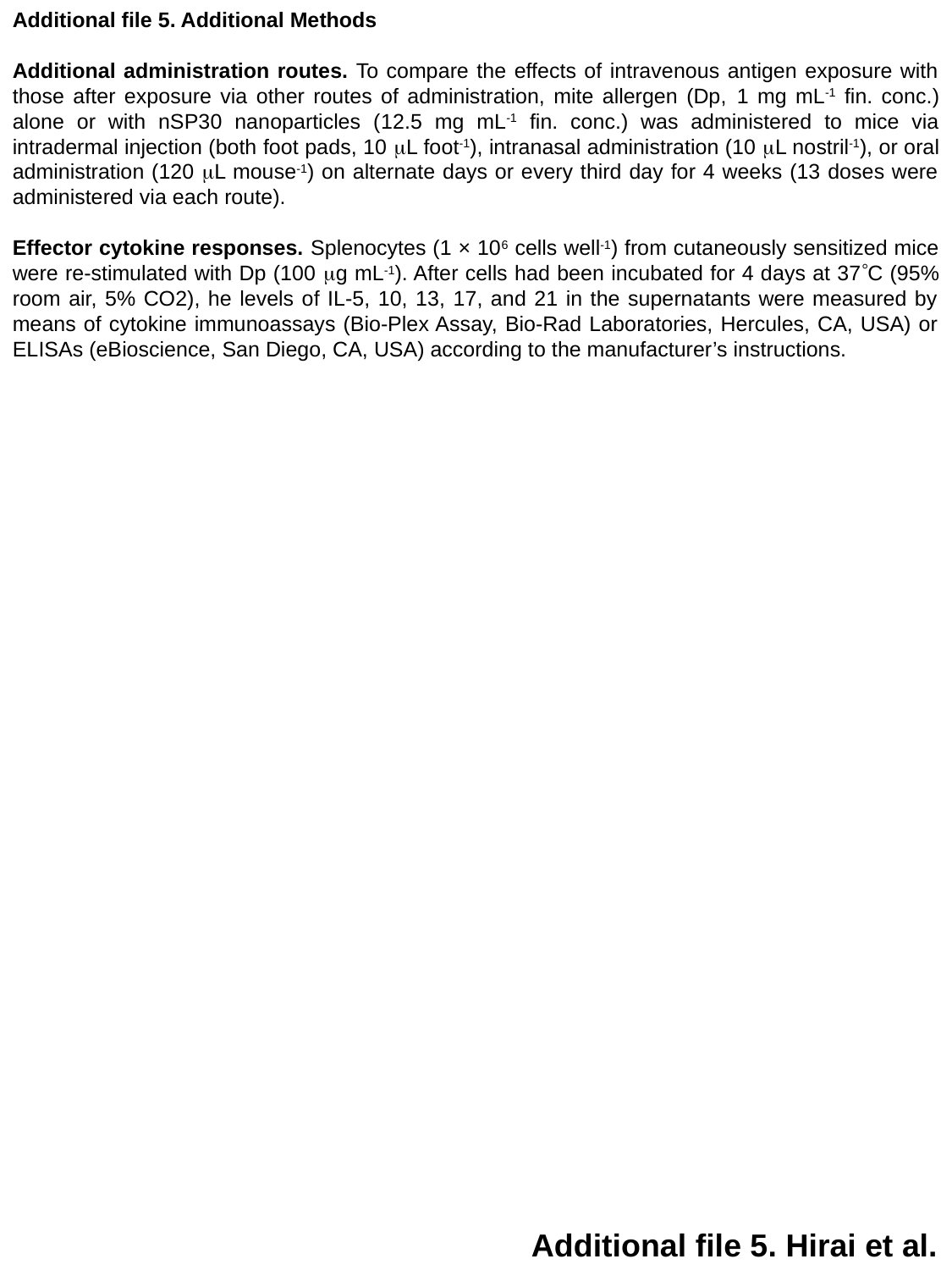

Additional file 5. Additional Methods
Additional administration routes. To compare the effects of intravenous antigen exposure with those after exposure via other routes of administration, mite allergen (Dp, 1 mg mL-1 fin. conc.) alone or with nSP30 nanoparticles (12.5 mg mL-1 fin. conc.) was administered to mice via intradermal injection (both foot pads, 10 μL foot-1), intranasal administration (10 μL nostril-1), or oral administration (120 μL mouse-1) on alternate days or every third day for 4 weeks (13 doses were administered via each route).
Effector cytokine responses. Splenocytes (1 × 10^6^ cells well-1) from cutaneously sensitized mice were re-stimulated with Dp (100 μg mL-1). After cells had been incubated for 4 days at 37°C (95% room air, 5% CO2), he levels of IL-5, 10, 13, 17, and 21 in the supernatants were measured by means of cytokine immunoassays (Bio-Plex Assay, Bio-Rad Laboratories, Hercules, CA, USA) or ELISAs (eBioscience, San Diego, CA, USA) according to the manufacturer’s instructions.
Additional file 5. Hirai et al.
